# Possible Role of Fish as Transport Hosts for *Dracunculus* spp. Larvae

**DOI:** 10.3201/eid2309.161931

**Published:** 2017-09

**Authors:** Christopher A. Cleveland, Mark L. Eberhard, Alec T. Thompson, Stephen J. Smith, Hubert Zirimwabagabo, Robert Bringolf, Michael J. Yabsley

**Affiliations:** University of Georgia, Athens, Georgia, USA (C.A. Cleveland, S.J. Smith, R. Bringolf, M.J. Yabsley);; Centers for Disease Control and Prevention, Atlanta, Georgia (M.L. Eberhard);; University of Oklahoma, Norman, Oklahoma, USA (A.T. Thompson); The Carter Center, Atlanta (H. Zirimwabagabo)

**Keywords:** zoonoses, parasites, dracunculiasis, Dracunculus medinensis, Dracunculus insignis, ferrets, fish, life cycle stages, Guinea worm disease, transport host, Chad, United States

## Abstract

To inform *Dracunculus medinensis* (Guinea worm) eradication efforts, we evaluated the role of fish as transport hosts for *Dracunculus* worms*.* Ferrets fed fish that had ingested infected copepods became infected, highlighting the importance of recommendations to cook fish, bury entrails, and prevent dogs from consuming raw fish and entrails.

The campaign to eradicate *Dracunculus medinensis* infection (Guinea worm disease) has helped 17 of 21 countries interrupt transmission (*1*). Endemic transmission of Guinea worm disease typically occurred via contamination of drinking water sources, resulting in community disease outbreaks. The absence of outbreaks of Guinea worm disease in Chad, coupled with increasing infections among domestic dogs in the transmission cycle (*2**–**6*), led to the hypothesis that transmission was occurring by different means. 

Previous work on *Dracunculus* and related spirurids indicates that paratenic hosts might be used to facilitate transmission (*7**,**8*). Recently, an experimental study showed *D. medinensis* worms could use tadpoles as paratenic hosts, and a naturally infected frog was detected in Chad (*9**,**10*). Few data exist on the potential role of fish as paratenic hosts; however, fish are suspected on the basis of epidemiologic data in Chad (*2*). Previous experimental attempts to infect Nile tilapia (*Oreochromis niloticus*) and fathead minnows (*Pimephales promelas*) with *D. medinensis* worms were not successful (*9*). However, in trials with *D. insignis* worms, 2 of 7 fish species exposed to high numbers of larvae became infected with low numbers of larvae (*11*). Collectively, these data suggest that fish are generally resistant to infection, need to ingest very high numbers of larvae to establish infection, or have variable species susceptibility.

Alternatively, it is possible that fish might ingest infected copepods and, if consumed by a host within short enough intervals, act as transport hosts. Our objective was to evaluate this potential role by allowing fish to ingest *Dracunculus* worm–infected copepods and then feed them to domestic ferrets to evaluate whether transmission could occur.

## The Study

In April 2016, we used first-stage larvae (L1) from gravid *D. insignis* worms from raccoons (*Procyon lotor*) in Georgia (USA) to infect colony-reared cyclopoid copepods (*7*). At 21 days postinfection, *D. insignis* larvae were examined to determine if they had developed to the infective third stage (i.e., trifid tail). If >25% of copepods were infected, they were used for transmission trials. Gravid *D. medinensis* worms were recovered from naturally infected dogs in Chad, and L1s were used to infect cyclopoid copepods collected from N’Djamena.

We then exposed groups of 5 Nile tilapia, fathead minnows, or mosquitofish (*Gambusia affinis*) to groups of 50 copepods (Table). We exposed fish to copepods for 3 hours in the first day of the *D. insignis* trial; on subsequent days, we exposed fish for 2 hours. All copepods provided to fish were consumed during exposures. Individual fish were removed, euthanized by exposure to neutral buffered tricaine methane sulfonate (MS-222) followed by pithing, and dissected. We observed digested copepods and free larvae in the intestine. 

**Table:** Results of ferret exposure trials with 3 different fish species exposed to copepods infected with *Dracunculus medinensis* or *D. insignis* worms

Fish species	*Dracunculus* sp.	No. fish consumed/no. offered*	Total no. copepods†	Days until euthanasia of ferret‡	*Dracunculus* infection status of ferret	No. worms recovered and sex§
Mosquitofish (*Gambusia affinis*)	*D. insignis*	28/30	300	91 and 134	–	0
	*D. medinensis*	30/30	300	77	+	1M/11F
Tilapia (*Oreochromis niloticus*)	*D. insignis*	27/30	300	91 and 134	+	6F¶
Fathead minnow (*Pimephales promelas*)	*D. insignis*	30/30	300	91 and 118	+	1M

*In groups of 5 fish/day for 6 days. †>25% of copepods infected. ‡The *D. insignis* worm–exposed ferrets have 2 entries for days until euthanasia because these animals were exposed to fish at 2 different time points with copepods infected with larvae from 2 different worms. §All worms were recovered from the subcutaneous tissues of the limbs. ¶Of these 6 female worms, 5 were gravid, indicating a male worm either was missed or had died before necropsy.

We fed the euthanized fish to laboratory-raised ferrets. If fish were not immediately ingested, we mixed the fish carcasses with cat food. Ferrets were fed fish in small batches. For *D. insignis* worms, we conducted exposures using copepods infected with larvae originating from 2 female worms at 2 different times (i.e., 5 fish per day for 3 days in April 2016 and another 5 fish per day for 3 days in July 2016, resulting in exposure to ≈300 copepods) (Table). Because there was only 1 *D. medinensis* worm, fewer *D. medinensis* worm–infected copepods were available. We exposed only 1 species of fish (mosquitofish). A ferret was given 5 fish per day for 6 days for a total of ≈300 copepods. (Table).

We maintained exposed ferrets for 77–134 days, then anesthetized and humanely euthanized them using 30 mg/kg ketamine followed by sodium pentobarbital. We necropsied the ferrets and examined the recovered *Dracunculus* worms to determine sex and whether females were mated or gravid. All animal procedures were reviewed and approved by the University of Georgia’s Institutional Animal Care and Use Committee (no. A2014 11–010).

Of the 3 ferrets we fed fish that had ingested *D. insignis* worm–infected copepods, 2 were infected (Table). One ferret was infected with 6 *D. insignis* females (5 gravid), the other with 1 male worm (Table). The 1 ferret fed fish exposed to *D. medinensis* worm–infected copepods became infected with 12 worms; female worms were mated but not gravid because of their young age (Table).

## Conclusions 

The infection of ferrets with *Dracunculus* spp. worms after consuming fish that had eaten infected copepods demonstrates a novel transmission route. The unprecedented increase in the number of *D. medinensis* worm infections in dogs in Chad suggests the potential role of aquatic paratenic hosts (*2**,**12*). Classically, paratenic hosts become infected and facilitate transmission by bridging a trophic level, maintaining long-term infections, or concentrating larger worm burdens in their tissues (*3*). We suggest that fish can serve the role of transport hosts because fish did not have disseminated *Dracunculus* worm infection develop in our initial trials (C.A. Cleveland, unpub. data). Because most cases of Guinea worm disease occur in areas known for intense artisanal fishing and residents’ dependence on fish protein, it is likely that fish and other aquatic animals play a role in transmission.

In 2014, preventive measures such as cooking fish thoroughly, burying fish entrails, and preventing dogs from consuming fish entrails were implemented in Chad. By May 2015, interventions were implemented in >50% of at-risk communities (*1*). Although limited, surveys for natural infections in fish from Chad’s Chari River have not detected *D. medinensis* larvae (*2*; C.A. Cleveland, unpub. data). However, our findings suggest that the proposed intervention strategies involving fish are relevant and should continue. It is unclear what happens in Chad to small fish caught by fishermen; the fish might be consumed whole without cooking or, more likely, are discarded where dogs could consume them. During previous surveys of fish for *D. medinensis* worms, large numbers of copepods were observed in their gastrointestinal tracts, supporting their potential role as transport hosts (C.A. Cleveland, unpub. data). Despite the interventions implemented in Chad, sporadic dog and human infections are still reported, suggesting a need for continued educational campaigns.

The recent report of a natural amphibian paratenic host, combined with the results of our study, indicates that the transmission of *D. medinensis* worms is not as simple as once believed (*10*). Despite the highly successful eradication campaign, 4 countries (South Sudan, Mali, Ethiopia, and Chad) still report endemic *D. medinensis* worm transmission. All 4 countries now report infections in dogs, so novel intervention and eradication strategies are needed.

Although our study showed that fish can transmit *Dracunculus* larvae to ferrets, many questions remain. For example, it is likely that different fish species feed on copepods at different rates and have different gastrointestinal tract transit times. This might also explain why individual exposure of mosquitofish to *D. medinensis* or *D. insignis* worms led to infection in only the *D. medinensis*–exposed ferrets; the *D. insignis*–exposed fish were fed to ferrets an hour later than *D. medinensis*–exposed fish. It is possible that mosquitofish transit material through the gastrointestinal tract faster than the other species. Alternatively, the ferrets may not have become infected simply because, as previous work has shown, not all ferrets exposed to *Dracunculus* worms become infected (*12*). Additional data are especially needed for those fish species that might be caught and ingested by humans or dogs in Guinea worm–endemic countries. Furthermore, 2 fish species retained *D. insignis* larvae in their tissues for 7–11 days, demonstrating the need for further experimental and field work on the role of fish as paratenic hosts for *Dracunculus* spp. worms (*6*).
